# Impact of patients´ age on short and long-term outcome after carotid endarterectomy and simultaneous coronary artery bypass grafting

**DOI:** 10.1186/s13019-019-0928-5

**Published:** 2019-06-15

**Authors:** Mona Salehi Ravesh, Rene Rusch, Christine Friedrich, Christoph Teickner, Rouven Berndt, Assad Haneya, Jochen Cremer, Thomas Pühler

**Affiliations:** 1 Department of Radiology and Neuroradiology, University Hospital Schleswig-Holstein Campus Kiel, Arnold-Heller-Street 3, Building 41, 24105 Kiel, Germany; 2 Department of Cardiovascular Surgery, University Hospital Schleswig-Holstein Campus Kiel, Arnold-Heller street 3, Building 18, Kiel, Germany

**Keywords:** Coronary artery bypass grafting, Carotid endarterectomy, Age

## Abstract

**Background:**

The purpose of this study was to investigate whether age has an effect on short and long-term outcome in patients who undergo simultaneous coronary artery bypass grafting (CABG) and carotid endarterectomy.

**Methods:**

From 2005 to 2017, 186 consecutive elective patients underwent CABG and synchronous endarterectomy at our institution. Patients were retrospectively classified according to age into 2 groups: patients above 70 years (elderly group: *n* = 97, 76.1 ± 3.9 years) and patients below 70 years (younger group: *n* = 89, 63.2 ± 4.8 years).

**Results:**

The European System for Cardiac Operative Risk Evaluation (EuroSCORE) II, 4.4% vs. 2.5%; *p* < 0.001) and Society of Thoracic Surgeons (STS) score (0.7% vs. 1.6%; *p* < 0.001) were significantly higher in the elderly group. Otherwise, there was no difference between the two groups concerning important preoperative risk factors or the intraoperative data. Postoperatively, the incidence of temporary dialysis was significantly higher in the elderly group (14.4% vs. 3.4%; *p* = 0.009). The rate of tracheotomy (16.5% vs. 2.2%; *p* = 0.001), of re-intubation (7.9% vs. 18.6%; *p* = 0.033) and drainage loss (600 ml vs. 800 ml; *p* = 0.035) was significantly higher in this elderly group. Neurological complications and 30-day mortality were comparable. Long-term survival was satisfactory for both groups. Nevertheless, 5-year survival rates (63% vs. 85%) were significantly lower in the elderly group (*p* = 0.003). Logistic regression analysis identified chronic obstructive pulmonary disease (COPD) and arrhythmia as significant risk factors for 30-day-mortality, but not age.

**Conclusions:**

CABG in combination with synchronous endarterectomy can also be performed with satisfactory results in elderly patients.

**Electronic supplementary material:**

The online version of this article (10.1186/s13019-019-0928-5) contains supplementary material, which is available to authorized users.

## Background

Coronary artery disease (CAD) is the most common type of heart disease and cause of mortality in the developed countries [1]. According to global and regional projections of mortality and burden of disease from 2002 to 2030, CAD will remain the leading cause of death in these countries for the next years to come [2]. In 2013, stroke was the second most common cause of death worldwide after ischemic heart disease [3]. Extracranial internal carotid artery stenosis is associated with around 8% of all ischemic strokes [4]. Significant coronary artery stenosis is a frequent additional finding in patients with repeat carotid artery stenosis (≥75%) [5]. Predictors of coincidence of CAD and carotid artery stenosis are advanced age, smoking, obesity, diabetes mellitus, arterial hypertension, and hyperlipidemia [6]. The presence of an additional carotid stenosis makes planning for the surgical treatment of CAD complicated. There are two surgical strategies (simultaneous and staged) for the treatment of concomitant carotid and coronary stenosis. On one hand, the staged surgical strategy is associated with a high risk of myocardial infarction if the carotid endarterectomy (CEA) or carotid artery stenting (CAS) is performed prior to coronary artery bypass grafting (CABG). On the other hand, this strategy is associated with an ischemic stroke if the CABG is carried out before the CEA/CAS [7, 8]. The period between the CEA/CAS and CABG is usually between 9 and 70 days [9]. The combined approach for CAE and CABG was reported for the first time in the 1970s [10, 11]. The simultaneous („same-day“) strategy for CAS and CABG was first presented in the multicenter and prospective SHARP study as a new successful treatment approach in 2009. In the simultaneous strategy, patients underwent CABG immediately after CAE or CAS. [12]. The optimal operative strategy for patients with concomitant carotid and coronary artery stenosis is still controversial and widely debated [13–15]. The CAS was introduced as a minimally invasive endovascular alternative to CEA, particularly for high-risk patients with an advanced age of > 80 years [16]. Recently published results from the CREST and ACT1 trial have demonstrated similar long-term outcomes for CAS and CEA surgical approaches with respect to the risk of stroke, myocardial infarction, or death [17, 18]. Apart from the surgical strategies employed for the treatment of coronary and carotid stenosis, many studies have shown a significantly greater postoperative risk of stroke and death in patients with an advanced age than in younger patients [13, 19, 20].

The purpose of this study was to investigate whether age has an effect on outcome in patients who undergo simultaneous coronary artery bypass grafting and carotid endarterectomy.

## Methods

### Patients and study design

In our center, 186 consecutive elective adult patients had a simultaneous CABG and CAE between January 2005 and December 2017. Patients were divided into two groups: younger than and equal to or older than 70 years. The younger group consisted of 89 patients (63.2 ± 4.8 years) and the elderly group of 97 patients (76.1 ± 3.9 years).

In framework of our standard clinical concept, the extracranial cardotid arteries of each patient is examined first using ultrasound, before a CABG operation. If there is a suspicious of carotid artery stenosis, the patient is additionally examined using computed tomography (CT) or magnetic resonance imaging (MRI). After the confirmation of the indication, the patient will prepared for the simoultanous surgical procedure.

The study population included all patients who underwent elective isolated CABG surgery with CAE. Exclusion criteria for this study were combined procedures or off-pump surgery.

All clinical data were collected prospectively on admission and during the in-hospital stay. We retrieved the data retrospectively by reviewing the hospital records. Primary end points were intraoperative and 30-day mortality, as well as long-term survival. Secondary end points were postoperative course (ventilation time, drainage loss, acute renal failure, neurologic complications). Patients were followed up directly in our out-patient clinic, seen by their general practitioner, or contacted directly by telephone or e mail.

Detailed information about the used surgical technique and statistical analysis in this work are available in the Additional file 1.

## Results

### Demographics and preoperative characteristics

Relevant demographics and preoperative data of patients are given in Table 1. The patients in the elderly group were significantly older than the patients in the younger group (76.1 ± 3.9 vs. 63.2 ± 4.8, *p* < 0.001). The logistic EuroScore II (4.4% vs. 2.5%; *p* < 0.001) and STS score (1.6 vs. 0.7; *p* < 0.001) were significantly higher in the elderly group. The median creatinine value in the elderly group was significantly higher (1.1 vs. 0.9 mg/dl, *p* < 0.003). There was no difference between the two groups concerning the preoperative risk factors or clinical presentation.

**Table 1:** Baseline and preoperative characteristics

Variable	Total number *N* = 186	Age < 70 years *N* = 89	Age ≥ 70 years *N* = 97	*p*-value
Age (years)	70.0 ± 7.8	63.2 ± 4.8	76.1 ± 3.9	**< 0.001**
	70.0 (64.8;76.3)	64.0 (60.0;67.0)	76.0 (73.0;79.0)	
Female gender, (%)	40 (21.5)	21 (23.6)	19 (19.6)	0.506
BMI (kg/m^2^)	27.7 ± 4.8	27.6 ± 4.4	27.8 ± 5.2	0.833
Logistic EuroScore ll (%)	3.4 (2.3;6.2)	2.5 (1.8;4.5)	4.4 (3.1;7.2)	**< 0.001**
STS-Score (%)	1.0 (0.6;1.8)	0.7 (0.5;0.9)	1.6 (1.0;2.5)	**< 0.001**
COPD, n (%)	23 (12.4)	12 (13.5)	11 (11.3)	0.657
Creatinine (mg/dl)	1.07 (0.84;1.21)	0.9 (0.8;1.1)	1.1 (0.9;1.3)	**0.003**
Dialysis, n (%)	3 (1.6)	1 (1.1)	2 (2.1)	0.622
Diabetes mellitus, (%)	61 (32.8)	31 (34.8)	30 (30.9)	0.571
IDDM, n (%)	25 (13.4)	13 (14.6)	12 (12.4)	0.655
Hyperlipidemia, (%)	137 (73.7)	67 (75.3)	70 (72.2)	0.630
Arterial hypertension, (%)	159 (85.5)	73 (82.0)	86 (88.7)	0.199
Pulmonary hypertension	12 (6.5)	4 (4.5)	8 (8.2)	0.298
PAVK, n (%)	54 (29.0)	28 (31.5)	26 (26.8)	0.485
Carotid stenosis, right side, (%)				
1 = < 50%	57 (30.6)	26 (29.2)	31 (32.0)	0.523
2 = 50–69%	13 (7.0)	8 (9.0)	5 (5.2)	
3 = 70–89%	63 (33.9)	27 (30.3)	36 (37.1)	
4= > 90%	53 (28.5)	28 (31.5)	25 (25.8)	
Carotid stenosis, left side, n (%)				
1 = < 50%	55 (29.6)	25 (28.1)	30 (30.9)	0.993
2 = 50–69%	16 (8.6)	8 (9.0)	8 (8.2)	
3 = 70–89%	71 (38.2)	34 (38.2)	37 (38.1)	
4 = > 90%	44 (23.7)	22 (24.7)	22 (22.7)	
Symptomatic carotid stenosis, n (%)	48 (25.8)	26 (29.2)	22 (22.7)	0.309
Neurological diseases, n (%)	10 (5.4)	7 (7.9)	3 (3.1)	0.198
Cerebral ischemia, n (%)				
TIA	6 (3.2)	4 (4.5)	2 (2.1)	–
PRIND	1 (0.5)	0	1 (1.0)	
Apoplexy	30 (16.1)	18 (20.2)	12 (12.4)	
Classification, n (%)				
One-vessel disease	7 (3.8)	5 (5.6)	2 (2.1)	–
Two-vessel disease	21 (11.3)	8 (9.0)	13 (13.4)	
Three-vessel disease	158 (84.9)	76 (85.4)	82 (84.5)	
Angina pectoris, n (%)	124 (66.7)	60 (67.4)	64 (66.0)	0.836
EF (%)	55.0 ± 16.3	56.8 ± 16.3	53.4 ± 16.2	0.264
Rhythm				
Atrial fibrillation, n (%)	25 (13.4)	3 (3.4)	22 (22.7)	–
Pacemaker, n (%)	4 (2.2)	1 (1.1)	3 (3.1)	
Acute myocardial infarction, n (%)	41 (22.2)	16 (18.0)	25 (26.0)	0.187
Previous heart surgery	9 (4.8)	6 (6.7)	3 (3.1)	0.315
Previous PCI, n (%)	35 (18.8)	18 (20.2%)	17 (17.5%)	0.638
CPR, n (%)	1 (0.5)	1 (1.1)	0	0.478

*BMI* body mass index, *COPD* chronic obstructive pulmonary disease, *CPR* cardiopulmonary resuscitation, *EF* ejection fraction, *IDDM* insulin-dependent diabetes mellitus, *PAVK* peripheral artery occlusive disease, *PCI* percutaneous coronary intervention, *PRIND* persistent reversible ischemic neurologic deficit, *STS score* society of thoracic surgeons score, *TIA* transient ischemia attack

The significant *p*-value are marked in bold

### Intraoperative data

The procedure time for the CEA in the younger group was significantly longer than that in the elderly group, but without clinical relevance (88 min vs. 80 min; *p* = 0.018). The intraoperatively administered number of red blood cell (RBC) units was higher in the elderly group, although this difference was not statistically significant (3.5 ± 2.3 vs. 2.3 ± 1.9, *p* = 0.052). Otherwise no significant differences were noted between the two groups with regard to intraoperative data. The number of distal anastomoses, the extracorporeal circulation time and the cross-clamp time were similar in both groups (Table 2).

**Table 2:** Operation and intraoperative findings

Variable	Total *N* = 186	Age < 70 years *N* = 89	Age ≥ 70 years *N* = 97	*p*-value
Urgency status of operations				
Elective, (%)	158 (84.9)	76 (85.4)	82 (84.5)	–
Urgent, (%)	24 (12.9)	11 (12.4)	13 (13.4)	
Emergency, (%)	4 (2.2)	2 (2.2)	2 (2.1)	
Operated carotid side				
1 = right side, (%)	91 (48.9)	48 (53.9)	43 (44.3)	0.191
2 = left side, (%)	95 (51.1)	41 (46.1)	54 (55.7)	
Carotid operation technique				
1 = Clamping, (%)	113 (60.8)	54 (60.7)	59 (60.8)	0.983
2 = Shunt, (%)	73 (39.2)	35 (39.3)	38 (39.2)	
Procedure time for CEA (min)	85.0 (61.5;180.0)	88.0 (70.0;215.0)	80.0 (60.0;101.0)	**0.018**
Procedure time for CABG (min)	271.0 (230.0;317)	271.0 (234.0;313.5)	272.0 (225.0;319.8)	0.844
Number of distal anastomoses	3.0 (3.0;4.0)	3.0 (3.0;4.0)	3.0 (2.0;4.0)	0.303
Bypass time (min)	126.4 ± 42.1	124.0 ± 41.0	128.6 ± 43.2	0.469
Aortic cross clamp time (min)	74.7 ± 35.0	70.1 ± 35.8	78.3 ± 34.1	0.157
RBC, units	3.0 ± 2.2	2.3 ± 1.9	3.5 ± 2.3	**0.052**
FFP, units	0.0 (0.0;0.0)	0.0 (0.0;0.0)	0.0 (0.0;0.0)	0.477
Platelet, units	0.0 (0.0;1.0)	0.0 (0.0;1.0)	0.0 (0.0;1.0)	0.431

*CABG* coronary artery bypass grafting, *CEA* carotid endarterectomy, *FFP* fresh frozen plasma, *RBC* red blood concentrate

The significant *p*-value are marked in bold

### Postoperative data

The incidence of postoperative temporary dialysis was significantly higher in the elderly group (14.4% vs. 3.4%; *p* = 0.009, Table 3). The 48-h drainage loss was significantly higher in the elderly group (800 [440; 1700] ml, vs. 600 [300; 1075] ml, *p* = 0.035). However, the postoperatively administered number of RBC units was similar in both groups. Pulmonary infections occurred more frequently in the elderly group (12.4% vs. 4.5%; *p* = 0.052). Consequently, the incidence rate of re-intubation (18.6% vs. 7.9%; *p* = 0.033) and tracheotomy (16.5% vs. 2.2%; *p* = 0.001) were significantly higher in the elderly group. Other factors determining ICU stay, such as postoperative neurological complications (6.2% vs. 3.4%; *p* = 0.501) or sternal wound infection (7.2% vs. 3.4%; *p* = 0.335), were comparable.

**Table 3:** Postoperative incidents and outcomes

Variable	Total *N* = 186	Age < 70 years *N* = 89	Age ≥ 70 years *N* = 97	*p*-value
Rhythm				
Atrial fibrillation (%)	18 (9.7)	5 (5.6)	13 (13.5)	–
Pacemaker (%)	8 (4.3)	4 (4.5)	4 (4.2)	
ICU (days)	2.0 (1.0;4.0)	1.0 (1.0;4.0)	2.0 (1.0;6.0)	0.122
Ventilation (hours)	17.0 (12.0;40.0)	17.0 (12.0;24.5)	17.5 (12.3;60.0)	0.290
Temporary dialysis, (%)	17 (9.1)	3 (3.4)	14 (14.4)	**0.009**
RBC, units	2.0 (2.0;4.0)	2.0 (2.0;4.0)	2.0 (2.0;4.0)	0.407
FFP, units	0.0 (0.0;4.0)	0.0 (0.0;2.0)	0.0 (0.0;4.0)	0.326
Platelet, units	0.0 (0.0;0.0)	0.0 (0.0;0.5)	0.0 (0.0;0.3)	0.990
Drainage blood (ml)	700.0 (400.0;1500.0)	600.0 (300.0;1075.0)	800.0 (440.0;1700.0)	**0.035**
Pulmonary infections, (%)	16 (8.6)	4 (4.5)	12 (12.4)	0.052
Perioperative myocardial infarction, (%)	8 (4.3)	1 (1.1)	7 (7.2)	0.066
Reexploration for bleeding, (%)	11 (5.9)	7 (7.9)	4 (4.1)	0.280
Sternal wound infection, (%)	9 (4.8)	4 (4.5)	5 (5.2)	1.000
Re-intubation, (%)	25 (13.4)	7 (7.9)	18 (18.6)	**0.033**
Tracheotomy, (%)	18 (9.7)	2 (2.2)	16 (16.5)	**0.001**
Stroke (CT proved), (%)	9 (4.8)	3 (3.4)	6 (6.2)	0.501
30d-MACCE, (%)	17 (9.4)	5 (5.8)	12 (12.8)	0.111
30-day mortality (%)	9 (5.1%)	2 (2.3%)	7 (7.6%)	0.171

*CT* computed tomography, *ECMO* extracorporeal membrane oxygenation, *FFP* fresh frozen plasma, *ICU* intensive care unit, *IMC* intermediate care station, *MACCE* major adverse cardiac and cerebrovascular events RBC: red blood concentrate

The significant *p*-value are marked in bold

The 30-day mortality in the elderly group in Table 3 was slightly higher than in the younger group, but not significantly (7.6% vs. 2.3%, *p* = 0.171). There was no difference between the two groups concerning 30-day major adverse cardiac and cerebrovascular events (MACCE, 12.8% vs. 5.8%, *p* = 0.111).

**Fig. 1:**
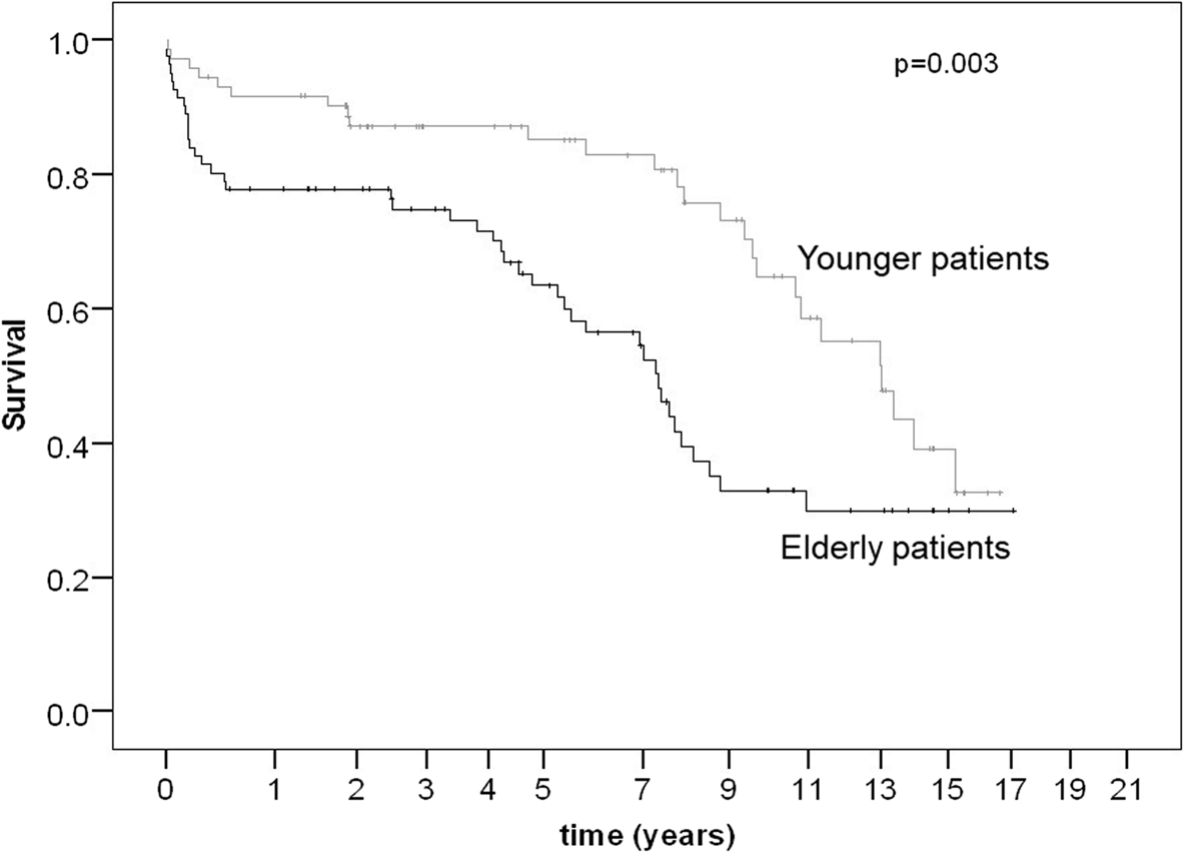
The estimated survival curves by Kaplan-Meier method

Long-term survival was satisfactory in both groups. Nevertheless, one-year (78% vs. 92%), 3-year (75% vs. 87%) and 5-year (63% vs. 85%) survival rates were significantly lower in the elderly group (*p* = 0.003) (Fig. 1). The Logistic regression analysis identified preoperative COPD and arrhythmia (atrial fibrillation or pacer) as significant risk factors for 30-day-mortality with odds ratios of 5.7 (CI 1.2–26.9) and 7.1 (CI 1.6–31.7), respectively (Table 4).

**Table 4:** Predictors for 30-day mortality

Variable	Odds Ratio	95% Confidence interval	*p*-value
COPD	5.684	1.202–26.878	0.028
Arrhythmia (AF or pacer)	7.147	1.612–31.692	0.010

## Discussion

In our single-center study, the effect of age on outcome in 186 patients who underwent simultaneous CABG and CAE was investigated. The patients were divided into two groups of younger than 70 and equal to or older than 70 years. The two groups were compared concerning their demographic, pre-, intra-, and postoperative data. There were no significant differences between the two patient groups concerning their pre- and intraoperative data, or their 30-day mortality and short-term major adverse cardiac and cerebrovascular events.

The optimal surgical approach (simultaneous or staged) for the treatment of patients with concomitant severe carotid and coronary stenosis is still the subject of controversial debate. In addition, with increasing age of the population, it is clinically relevant to clarify whether the postoperative risk of stroke and death in patients of an advanced age is higher than in younger patients.

In a larger analysis, Brott et al. . [17] evaluated the outcomes of 2502 patients at 117 centers within the framework of the CREST study every 6 months for up to 10 years. These patients (69.0 ± 8.9 years) had been randomly assigned to stenting or endarterectomy. Brott et al. did not find a significant difference between patient groups with respect to the risk of periprocedural stroke, myocardial infarction, or death and subsequent ipsilateral stroke. The rate of postprocedural ipsilateral stroke also did not differ between groups.

Feldman and colleagues [13] compared trends and outcomes of three approaches to carotid revascularization in the CABG population when performed during the same hospitalization: 1) combined CABG and CEA, 2) staged CEA and CABG, and 3) staged CAS and CABG. A total of 22,501 patients were included in this study. 15% of these patients were equal to/older than 80 years. A higher number of patients (15,402, 68.4%) underwent combined CABG and CAE, followed by staged CABG and CEA (6297, 28.0%), and staged CABG and CAS (802, 3.6%). The risk of stroke was lower in patients from the first and second groups compared with patients from the third group. The adjusted risk of death or stroke was similar in the 3 groups.

Sharma et al. [14] performed a meta-analysis of 12 studies comparing early outcomes of synchronous and staged approach of CABG and CAE. In these studies, a total of 17,469 and 7552 patients were included for the combined and staged approaches, respectively. The investigated endpoints were early mortality, major stroke, and major postoperative morbidity, myocardial infarction and stroke, and combined early mortality or stroke. Early events were compared using pooled estimates of risk ratios (random effects model) utilizing the inverse-variance method. The pooled analysis revealed no difference in early mortality (*p* = 0.27), postoperative stroke (*p* = 0.07), combined early mortality or stroke (*p* = 0.11), and combined endpoint of myocardial infarction or stroke (*p* = 0.2) between the two approaches.

The results of Brott et al., Feldman and Colleagues and Sharma et al. concerning the risk of periprocedural stroke, myocardial infarction are in line with our presented results.

In a retrospective single-center study, Wang et al. [21] reviewed the clinical data of octogenarians and younger patients to explore the association between age and outcome. Wang et al. reported that octogenarians are increasingly referred for elective cardiac surgery with more combined procedures (valve plus CABG or multiple valves) compared with younger patients (*p* < 0.001). The 30-day, 1-year and 5-year mortalities for octogenarians were 3.7, 10.8 and 29.0%, respectively. The octogenarians had higher adjusted 30-day (*p* = 0.018) and 1-year mortality (*p* < 0.001) compared to the younger group. Octogenarians had longer post-operative stays in ICU and hospital, and higher rates of ICU readmission (*p* < 0.001). After multi-variable adjustment, an age of older than or equal to 80 years was an independent predictor of death at 30 days and 1 year. In contrast to Wang et al., we did not find any differences between our patient groups concerning the post-operative stays in ICU and hospital, and also 30-day mortalities. But the one-year, 3-year and 5-year survival rates were significantly lower in our elderly group.

Alexander et al. [22] examined the predictors of in-hospital mortality in octogenarians, compared with the predictors in younger patients, who underwent cardiac surgery at 22 centers. Alexander et al. reported that octogenarians undergoing cardiac surgery had fewer comorbid illnesses, but higher disease severity and surgical urgency than younger patients. Octogenarians had significantly higher in-hospital mortality after cardiac surgery than younger patients: isolated CABG (8.1% vs. 3.0%), CABG and aortic valve replacement (10.1% vs. 7.9%), CABG and mitral valve replacement (19.6% vs. 12.2%). In addition, octogenarians had twice the incidence of postoperative stroke and renal failure. The preoperative clinical factors predicting CABG mortality in the very elderly were quite similar to those for younger patients. Of note, elderly patients without significant comorbidity had lower in-hospital mortality rates after CABG (4.2%) compared to those after combined CABG with aortic valve replacement (7%) and after combined CABG with mitral valve replacement (18.2%). Our elderly patient group showed a significant higher temporary dialysis, Drainage blood, re-intubation, and tracheotomy. Our data confirm the results of Alexader et al. regarding higher disease severity and surgical urgency in their elderly patient group.

Ohira et al. [23] investigated the relationship between age and both short- and long-term outcomes after off-pump CABG. They divided the patients into 3 groups: aged < 65 years (young), 65–74 years (early elderly), and > 75 years (late elderly), and retrospectively analyzed their clinical data. In-hospital mortality rates were similar among the groups. In logistic regression analysis, the risk factor for predicting major complications was the New York Heart Association (NYHA) classification (*p* = 0.001), and not age and preoperative myocardial infarction. The 10-year estimated rates free from cardiac death and cardiac events were not significantly different among the groups. In multivariate Cox models, independent risk factors predicting cardiac events were the NYHA classification, and ejection fraction, but not age. Ohira et al. reported that neither short- nor long-term cardiac outcomes after off-pump CABG are influenced by age at surgery.

## Conclusions

In our study, CABG in combination with synchronous endarterectomy can be performed with satisfactory results, especially in the specific high-risk subgroup of patients of advanced age. The multivariate logistic regression analysis of clinically relevant parameters indicated that there is no significant effect of age on outcome in patients who undergo simultaneous CABG and CAE with single anesthesia. Based on our current results, we would recommend this surgical approach. However, further prospective, multi-center, and randomized clinical studies with a larger group of patients are required to investigate in detail the effect of age on patient outcome.

### Limitations

The presented data were retrospective from a single center, and the sample size remains small.

## Additional file

Additional file 1: Extended material and methods section. (DOCX 25 kb)

## Data Availability

The dataset analyzed during the current study may be available from the authors on reasonable request.

## Abbreviations

CABG: Coronary artery bypass grafting
CAD: Coronary artery disease
CAS: Carotid artery stenting
CEA: Carotid endarterectomy
COPD: Chronic obstructive pulmonary disease
NYHA: New York Heart Association
