# Supplementary material for: Impact of patients´ age on short and long-term outcome after carotid endarterectomy and simultaneous coronary artery bypass grafting
Source: J Cardiothorac Surg. 2019 Jun 15;14:109. doi: 10.1186/s13019-019-0928-5 (PMC6570883; doi:10.1186/s13019-019-0928-5)
Supplement: Supplementary file 1 — Extended material and methods section. (DOCX 25 kb) [file 13019_2019_928_MOESM1_ESM.docx]

**Material and Methods**

*Surgical technique*

All of the carotid surgery was performed in general anesthesia directly before the CABG. After exposition of the common (CCA) and internal (ICA) carotid artery, two vascular loops were doubly passed around each to control in- and backflow of the vessels before temporary or instant clamping. Intravenous heparin was administered beforehand. In case of using a shunt, the ICA was incised in the distal intact arterial wall, and the distal end of the shunt was inserted and fixed by ligation with a vessel loop. The shunt was filled with blood retrograde and inserted into the CCA, and was also fixed with a double ligation with the vessel loop after careful retrograde de-airing. A longitudinal incision was made from the distal shunt site to the proximal shunt site; the intraluminal atheroma was exposed and fully dissected. After the endarterectomy, the arteriotomy was closed using a bovine pericardial patch and a 6/0 polypropylene running suture. The shunt was removed before completion of the suture after careful retro- and antegrade flushing and sialine purging of the dissected area. Every patient then received a small vacuum drainage through a separate small incision. Drainage was opened immediately after surgery and was removed on postoperative day two if no bleeding was present.

Median sternotomy was applied in all patients. Usually, the left internal thoracic artery (LITA) was harvested while the saphenous vein (SVG) and radial artery (RA) were prepared. After opening of the pericardium, extracorporeal circulation was installed. Mild hypothermia (34°C) was employed. Myocardial protection was obtained by antegrade application of cold blood cardioplegic solution. Distal anastomoses were accomplished in a standard fashion using prolene 8-0. The surgeries were performed by a number of different surgeons.

*Statistical analysis*

Statistical analysis was performed with SPSS 18.0 (SPSS Inc., Chicago, IL, USA). Descriptive statistics were used to describe patient characteristics throughout the study. Means and standard deviations were computed for normally distributed continuous variables, whereas medians and interquartile ranges were used to describe non-normally distributed continuous data. The student's t test was performed for comparison of normally distributed data and the Mann-Whitney U test was used for non-normally distributed data. Categorical variables are presented as frequency distributions (n) and simple percentages (%). A univariate comparison between the groups for categorical variables was performed using the χ² and the Fisher’s exact test when appropriate. Multiple logistic regression was performed to determine the relative impact (adjusted odds ratio, OR) of the preoperative variables age ≥ 70 years, female gender, COPD, myocardial infarction within 30 days and arrhythmia (atrial fibrillation or pacer) prior to surgery on 30-day mortality. The estimated survival was determined on the basis of survival curves using the Kaplan–Meier method and compared by using the log-rank test. Statistical significance was assumed at a p-value of ≤ 0.05. Data were analysed with IBM SPSS Statistics for Windows (Version 23.0).
